# Medicine to Kill, Medicine to Heal

**DOI:** 10.3201/eid0802.01-0514

**Published:** 2002-02

**Authors:** Neil Shulman, Zoe Haugo

[REMOVED ADVANCE FIELD] Recent bioterrorism events invite speculation on the nature and extent of human capacity for destructive as well as constructive behavior. Drawing a line between those who create suffering to further their cause and those who strive to alleviate suffering regardless of the cause is fair, though the dichotomy may foster intolerance and further polarize the opposition. 

 On the one hand, a shortage of open-mindedness and understanding compromises humans’ ability to exist in a secure state of peace. A single variable or difference becomes a huge point of contention. Religion has polarized Jews and Muslims in the Middle East, Protestants and Catholics in Ireland, Hindus and Muslims in India; tribal differences decimated Hutus and Tutsis in Rwanda; ideological feuds pounded Cambodia; and skin-color prejudice continues to inflame many countries. A leader can invoke a group of white humans or yellow humans to kill a group of black humans or red humans, but a single white cat could never convince a group of white cats to go out and kill a group of yellow cats. The brains of other animals are generally not wired in this way.

 On the other hand, an enormous capacity for compassion drives countless organizations to reduce suffering, eradicate disease, and promote the common good. Departments of public health, for example, join together throughout the world to fight epidemics. Workers from all countries, groups, and belief systems unite to stamp out the diseases that plague us. The road is long, and the breakthroughs are few. Many have risked and lost their lives in the dangerous pursuit of infectious-disease elimination. Disease prevention and control activities deemphasize ethnic differences and acknowledge the value of all human life. Although many public health workers are involved in laboratory research, disease surveillance, and the technical aspects of human health, they remain focused on the goal of their endeavors, the common good. 

 To prevent humans from harming each other and encourage them to work for the common good, perhaps we can "infect" the world with the value system of public health workers and nurture the circumstances of their psychological make-up. What is it that drives people to choose eliminating disease, improving public health, and extending or saving lives over the deliberate introduction of disease and destruction? Public health research should expand to address the psychodynamics of violent temper, corrosive anger, the use of disease as a weapon of war, and the circumstances and environments that promote them.

 If only, in temporary suspension of disbelief, life could imitate science fiction, humans would set aside their differences to fight a common enemy: disease not aliens.

